# Hepatitis Management in Saudi Arabia: Trends, Prevention, and Key Interventions (2016–2025)

**DOI:** 10.3390/medicina61091509

**Published:** 2025-08-22

**Authors:** Majed A. Ryani

**Affiliations:** Department of Family and Community Medicine, Faculty of Medicine, Jazan University, Jazan 45142, Saudi Arabia; majedryani@gmail.com

**Keywords:** hepatitis, Saudi Arabia, epidemiology, prevention, vaccination, treatment policies, public health

## Abstract

*Background*: Hepatitis presents a major health and economic challenge in Saudi Arabia, necessitating insight into its epidemiology, risk factors, and control measures. This review aims to synthesize current evidence on the epidemiology, risk factors, and prevention strategies for viral hepatitis in Saudi Arabia. It evaluates the effectiveness of existing interventions and proposes data-driven approaches to advance national hepatitis elimination goals. *Methods*: This study reviewed data from 2016 to 2024, sourced from PubMed, Google Scholar, ResearchGate, and ScienceDirect, focusing on hepatitis epidemiology and prevention in Saudi Arabia. Studies relevant to Saudi-specific trends and prevention strategies were included. *Results*: Saudi Arabia has achieved significant reductions in viral hepatitis prevalence, notably HBV (1.3%) due to universal infant vaccination (98% coverage), and HCV (0.124%) through the Saudi National Hepatitis Program (SNHP), which provides free DAAs (95% cure rate) and has screened 5 million people. However, challenges persist: HAV susceptibility is rising in adults (seroprevalence 33.1%), HDV affects 7.7% of HBV patients, and key risk factors include socioeconomic disparities (higher HAV/HEV in rural/low-income areas), intravenous drug use (30–50% of HCV cases), unsafe medical/cultural practices (e.g., Hijama), and limited healthcare access for migrants/rural populations. While interventions like water sanitation initiatives (58% HAV decline) and prenatal screening are effective, advancing elimination goals requires addressing gaps in HDV/HEV surveillance, outdated seroprevalence data, equitable treatment access (35% lower in rural areas), stigma reduction, and targeted strategies for high-risk groups to meet WHO 2030 targets. *Conclusions*: Saudi Arabia has made significant progress in hepatitis control through vaccination and public health efforts, but challenges persist. Strengthening healthcare systems, improving community engagement, and ensuring equitable access are key to sustaining elimination efforts.

## 1. Introduction

Viral hepatitis, characterized by liver inflammation, remains a major global health challenge, with significant variations in transmission, clinical outcomes, and public health impact. In Saudi Arabia, the epidemiology of viral hepatitis—encompassing hepatitis A (HAV), B (HBV), C (HCV), D (HDV), and E (HEV)—reflects both progress and persistent challenges shaped by demographic shifts, vaccination programs, and emerging risk factors. While notable successes, such as the dramatic decline in HBV prevalence due to universal infant vaccination, demonstrate the effectiveness of public health interventions, gaps in surveillance, evolving transmission dynamics, and understudied co-infections like HDV continue to complicate elimination efforts [1].

Historically, HAV was highly endemic in Saudi Arabia, particularly in regions with inadequate sanitation, with most infections occurring in childhood. However, improved living standards have reduced early-life exposure, leading to a growing population of susceptible adolescents and adults. Recent seroprevalence data show that anti-HAV IgG prevalence is only 33.1%, raising concern over the increased risk of severe outcomes, such as fulminant hepatitis, particularly in older age groups. This suggests that merely one-third of the population possesses immunity. In comparison, a previous study reported intermediate endemicity, with anti-HAV IgG rates of approximately 50% among adolescents and young adults, indicating a shrinking immune population [2]. This represents a decline compared to previous decades, likely due to improved sanitation and reduced natural exposure in early childhood. While this reflects progress in public health infrastructure, it also raises concerns about increasing susceptibility in older children and adults. Outbreaks could become more frequent in non-immune populations, necessitating targeted vaccination strategies, particularly for high-risk groups. This shift underscores the need for targeted vaccination strategies, especially for high-risk populations such as pilgrims and travelers [3]. Approximately 70% of infected adults develop symptoms, including jaundice. In contrast, only 30% of children younger than six years of age develop symptoms, which usually are nonspecific and flu-like without jaundice [4].

Unlike HAV, which is primarily transmitted through contaminated food and water, HBV spreads via blood and bodily fluids, making its epidemiology and prevention strategies distinct yet equally critical for public health planning. Its prevalence has significantly declined to an estimated 1.3%, largely due to the successful nationwide infant vaccination program introduced in 1989. A key complicating factor, however, is HDV co-infection, affecting 5–20% of HBV cases and markedly accelerating liver disease progression, with a heightened risk of cirrhosis and hepatocellular carcinoma (HCC). The burden is particularly pronounced in high-risk groups such as healthcare workers, hemodialysis patients, and expatriates from high-endemic regions [5]. These patterns underscore the importance of integrating enhanced HDV screening and targeted management into HBV care programs [6].

On the other hand, HCV prevalence remains relatively low (0.124% overall), with minimal perinatal transmission but persistent risks from unscreened blood transfusions (before 1990) and increasing concerns related to intravenous drug use (IDU). HEV is primarily waterborne, and its transmission is closely linked to environmental and sanitation factors. High-density gatherings and limited access to adequate sanitation can facilitate HEV spread, increasing the risk of outbreaks, particularly during mass events such as the Hajj pilgrimage. The dynamic population structure includes a large expatriate workforce and millions of annual pilgrims, which further complicates transmission patterns, necessitating robust surveillance and tailored prevention strategies [7].

Despite existing interventions, critical research gaps hinder optimal hepatitis control. There is a lack of recent nationwide seroprevalence data, particularly for HCV, HDV, and HEV, as well as insufficient exploration of the impact of migration and travel on disease burden. Additionally, emerging challenges such as drug-resistant viral strains and inadequate HDV surveillance require urgent attention [8]. Addressing these gaps is essential to refining national hepatitis policies and strengthening elimination efforts.

Therefore, this review aims to consolidate current evidence on the epidemiology, trends, and risk factors of viral hepatitis (HAV, HBV, HCV, HDV, and HEV) in Saudi Arabia, evaluating the effectiveness of existing prevention strategies, particularly the HBV vaccination program. It seeks to identify persistent challenges, including understudied co-infections and emerging risk factors like IDU, while proposing evidence-based strategies to enhance surveillance, prevention, and control.

## 2. Methodology

This study constitutes the narrative literature review examining the epidemiology, prevention strategies, and policy interventions related to viral hepatitis (HAV, HBV, HCV, HDV, and HEV) in Saudi Arabia. Data were extracted from peer-reviewed studies, published between 2016 and August 2025, sourced from authoritative databases including PubMed, PubMed Central, Google Scholar, ScienceDirect, and ResearchGate. To maintain focus, only studies primarily addressing Saudi Arabia were included, while regional studies from the broader Middle East were considered only if they provided directly applicable comparative insights.

The included studies comprised randomized controlled trials (RCTs), cohort studies, cross-sectional surveys, systematic reviews, and national surveillance reports addressing hepatitis prevalence, transmission dynamics, vaccination coverage, or prevention policies in Saudi Arabia. Studies were excluded if they did not focus on viral hepatitis (types A–E), lacked relevant epidemiological or policy-related data, or were limited to case reports, editorials, or non-peer-reviewed articles.

A structured search strategy was employed, utilizing targeted keywords such as “hepatitis prevalence Saudi Arabia,” “HBV vaccination policies KSA,” “HCV screening programs Saudi Arabia,” and “viral hepatitis prevention strategies Middle East.” Boolean operators were applied to refine the search, along with secondary filters restricting results to studies published between 2016 and 2025, written in English or Arabic, and categorized as epidemiological surveys, clinical trials, or policy analyses. Database-specific filters, including “human studies” and “full text available,” were also used to enhance precision.

Data extraction focused on key variables such as prevalence rates, vaccination coverage, risk factors, temporal trends, and policy recommendations. The synthesis method incorporated both quantitative and qualitative approaches: quantitative analysis involved pooled estimates of hepatitis prevalence using descriptive statistics where comparable, performed with Ms. Excel Version 16.99.2. while qualitative thematic synthesis organized policy and intervention findings into key themes such as “vaccination success” and “diagnostic gaps.”

Some methodological nuances included the limited consideration of real-time policy changes or outbreaks occurring after the search period. Additionally, variability in study designs and reporting methods also introduced heterogeneity, which limited the scope of some pooled analyses.

## 3. Results and Discussion

### 3.1. Epidemiology of Hepatitis in Saudi Arabia

In Saudi Arabia, the incidence and prevalence of viral hepatitis—including HAV, HBV, and HCV—have significantly declined over recent decades, largely due to sustained public health initiatives such as comprehensive vaccination campaigns, improved sanitation, and enhanced screening and treatment strategies.

#### 3.1.1. Hepatitis A

Hepatitis A (HAV) occurs sporadically worldwide and follows a cyclical recurrence pattern, with approximately 1.5 million clinical cases reported annually. In high-income countries, it accounts for 20–25% of viral hepatitis cases, though this proportion is higher in low- and middle-income nations due to historically poorer sanitation and hygiene. Socioeconomic improvements including increased income, better access to clean water, and vaccination uptake have significantly influenced HAV epidemiology over the past two decades (Table 1).

**Figure 1:**
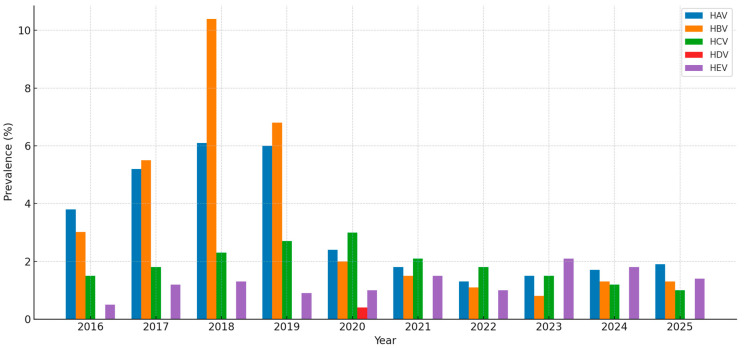
Prevalence of hepatitis in Saudi Arabia (2016−2025).

**Figure 2:**
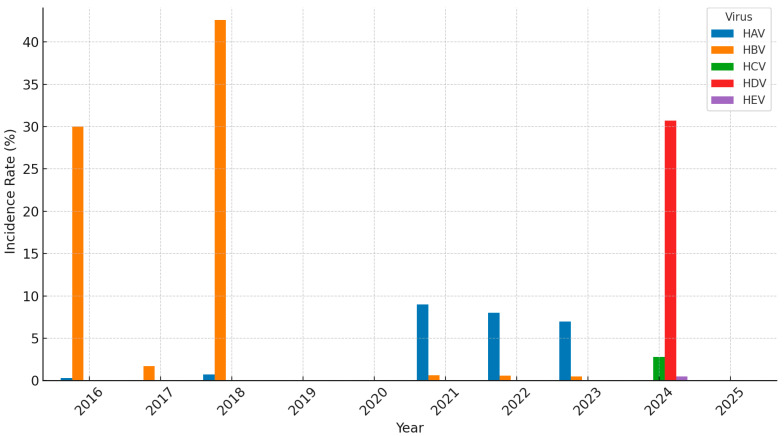
Incidence of hepatitis in Saudi Arabia (2016−2025).

Hepatitis A incidence in Saudi Arabia has shown considerable fluctuations over the years. Blood donor data reflect notable variations, with rates of 0.32% in 2016 rising to 0.74% in 2018 [10], surging sharply to 9% in 2020, and then gradually declining to 8% in 2021 and 7% in 2022 [11], highlighting occasional rebounds despite an overall downward trend (Figure 1). Similarly, MOH data showed fluctuation, 157 cases of HCV were reported in 2023, and rates declined from 0.84% in 2019 to a low of 0.09% in 2022, before rising again to 0.47% in 2023 [9]. This striking decline mirrors the country’s progress in sanitation, food safety, and vaccination efforts, while the earlier peak reflects a widespread exposure in previous years (Figure 2).

Seroprevalence studies reveal regional and age-related differences. In Riyadh, 45% seroprevalence was found by age 11 [12], while Arif et al. reported 24.7% by age 12, both indicating intermediate endemics [13]. A study in Jeddah, Western Saudi Arabia, revealed a 28.7% seropositivity rate among schoolchildren [14]. In contrast, high HAV endemicity in the Eastern region (Dammam) was already elevated among school-aged children and increased with age [15]. The higher seroprevalence observed in older age groups and less developed areas reflects historical exposure during times of limited sanitation and healthcare infrastructure. Seasonal variations were observed, with HAV cases peaking in March and September, coinciding with the post-winter indoor congregation and post-summer return to schools or workplaces, facilitating fecal–oral transmission [16].

Seasonal variations were observed, with HAV cases peaking in March and September. These surges coincided with transitional periods—post-winter indoor congregation and post-summer return to schools or workplaces—facilitating fecal–oral transmission [17].

#### 3.1.2. Hepatitis B

The World Health Organization (WHO) estimated that around 254 million individuals were living with chronic hepatitis B globally in 2022, with approximately 1.2 million new chronic cases occurring each year [18]. According to the 2017 WHO Global Hepatitis Report, the estimated prevalence of HBV in the Eastern Mediterranean region—which includes Saudi Arabia—was around 3.3%. However, in this region, there are an estimated 18 million people with HBV in 2024; the United Arab Emirates (UAE) is a low-endemic region with a prevalence of <1% [19]. Furthermore, MOH reported 7658 cases of HCV in 2023 [9].

Globally, the highest HBV rates found were 6.2% in the Western Pacific and 6.1% in Africa. A 2016 blood donor review revealed that the Middle East had an average HBV prevalence of 1.62%, with Saudi Arabia recording a higher rate of 3.02% compared to 0.67% in Iraq and Iran 0.58% in Iran [16]. However, it should be noted that blood donor data may not fully represent the general population, potentially leading to underestimation due to donor screening or overestimation if high-risk groups are overrepresented. Saudi Arabia has seen a significant decline in HBV prevalence since the introduction of universal infant vaccination in 1990. A study by Al Faleh et al. (2015) documented a drop from ~8% in the 1980s to just 1.3% in vaccinated cohorts in Riyadh, highlighting the success of immunization programs [20].

Reflecting these historical trends, more recent data demonstrate a continuing decline in HBV prevalence in Saudi Arabia. In 2016, the prevalence was about 3.02% [18]; however, after two years, it increased to 10.4% in 2018 [21], followed by a progressive reduction to 0.8% in 2024 [22,23,24]. A minor rise to 1.3% in 2025 suggests an ongoing low-level transmission [25], highlighting the continued importance of maintaining high vaccination coverage and routine screening programs (Figure 1).

HBV incidence also exhibited considerable variability in surveillance and blood donor data. In 2016, the rate was notably high at 30%, which increased to 42.60% in 2018 [26]. In 2020, the prevalence declined sharply to 0.63%, followed by 0.60% in 2021 and 0.50% in 2022 [26] (Figure 2).

Despite these encouraging trends, significant geographic variability persists within the country. Regional data (2021) from the Saudi Ministry of Health (MOH) revealed that the highest crude incidence rates (CIR) were observed in Qunfudah, Jeddah, Tabuk, and Taif (25–29 per 100,000), while the lowest rates were reported in Hail, Qurayyat, Jouf, and Hafr Al Baten (1–4 per 100,000). Similarly, El Beltagy et al. noted interregional differences in hepatitis B surface antigen (HBsAg) prevalence among blood donors, with higher rates in the Eastern region (3.24%) compared to Riyadh (1.5%). However, blood donors may not fully represent the general population, as they are typically healthier and prescreened, potentially underestimating the true HBV prevalence in these regions [27].

Age-specific data reveal distinct trends in HBV incidence rates [28]. Children aged 0–14 years show the lowest incidence of about 0.3–0.8 per 100,000, while adults aged 15–44 experience a moderate rate of about ~14 per 100,000. The highest incidence occurs in individuals aged 45 and above reported at ~30 per 100,000. Additionally, gender disparities exist, with Saudi males having a 1.4 times higher HBV incidence than females. Among non-Saudi populations, this disparity increases, with males exhibiting a 2.2-fold higher rate. Supporting this trend, data from Saudi Aramco Medical Services in the Eastern Province (2021) indicate that males had a 1.8 times greater HBV risk than females per 100,000 people due to greater exposure to risk factors like occupational hazards, tattoos, and high-risk behaviors. Cultural and social norms may also limit female exposure, reducing their infection rates [29] (Table 1).

In the UAE, public awareness of HBV remains limited. A 2024 study found that only 53% of the general population were aware of HBV, and merely 21% understood its nature and modes of transmission. This underscores the need for continued education and public health interventions alongside vaccination and screening programs to further reduce the HBV burden in the region.

#### 3.1.3. Hepatitis C

The WHO (2024) estimates that the Eastern Mediterranean region is a hotspot for HCV, with approximately 12 million people infected, representing the highest prevalence among all WHO regions [30]. Acknowledging this, the Saudi MOH launched a national elimination plan aiming to cut new HCV cases by 90% and related deaths by 65% by 2030 [31], and it reported 12733 cases of HCV in 2023 [9]. The prevalence of HCV in Saudi Arabia fluctuated significantly over recent years, with a sharp increase from 4. 24% in 2017 to a peak of 22.63% in 2020 [12], likely reflecting low-level screening initiatives. The following years showed a steady decline, with the prevalence falling to 12.09% in 2021, 12.34% in 2022 [29], and dropping dramatically to 0.40%, 0.23%, and 0.09% in 2023 [32], 2024 [24], and 2025 [33], respectively, indicating the impact of effective treatment strategies and public health interventions (Figure 1).

HCV remained rare throughout other periods. It was detected in 2020, with an incidence rate of 0.01%, which persisted in 2021 [34]. It rose sharply in 2024 to 2.80% [35] (Figure 2). In a study conducted in 2023, the high incidence of HCV was linked to several factors, particularly the lowest HCV testing rate and comparatively limited knowledge among its residents compared to other regions [36]. A recent Cureus survey (2019–2022) reported a 56.9% reduction in acute HCV notifications, from 9.94 to 4.29 per 100,000, with adult males and individuals ≥45 years remaining the most affected groups. In high-risk cohorts, such as IDU, prevalence is alarmingly higher (38–69%), with genotype 1b predominating.

Age trends show an increasing seroprevalence with age: from 4.49% (<15 years) to 15% (45–54 years), though hospital-based or mixed datasets may inflate estimates. Government data confirm a low seropositivity in children of about 0.01% rising to 0.20% in adults. Riyadh-specific cross-sectional data (2019–2022) revealed a lifetime HCV seroprevalence of 5.8%, associated with the male gender, marital status, diabetes, and hypertension. These seroprevalence data indicate past or lifetime exposure to the virus, not necessarily current or active infection, particularly from hospital-based or mixed datasets, especially in populations with previously cleared infections or successful treatment. Jeddah remained the most heavily burdened region, accounting for 35.8% of reported cases, though it experienced a 29.3% decline over the study period [34] (Table 1).

#### 3.1.4. Other Hepatitis Types

Hepatitis D virus (HDV), a defective virus requiring HBV coinfection, affects an estimated 7.7% of chronic HBV patients in Saudi Arabia (reported in 2020), contributing to more aggressive liver disease progression [37]. Whereas in 2025, the incidence of HDV was reported as 30.70% [6]. Despite the success of HBV vaccination, HDV remains understudied, and limited data highlight the need for consistent surveillance to better assess co-infection burdens among HBV cases. Although HEV typically results in acute, self-limiting infections, pregnant women represent a high-risk group, facing severe outcomes such as fulminant hepatitis, miscarriage, and maternal mortality—with case fatality rates reported as high as 20–30% during certain outbreaks.

Saudi MOH reported 15 cases of HEV in 2023 [9]. Moreover, HEV prevalence has varied widely in the region, recorded at 20% in 2017 [38], dropping to 4.3% in 2022 [39], before spiking again to 23.8% in 2023 [40], likely reflecting outbreak dynamics, environmental factors, and population movement, particularly during mass gatherings (Figure 1). HEV was undetected in most study years, with an incidence rate of 0.50% in 2022 [41] (Figure 2). Despite these fluctuations, the true burden of HEV remains uncertain due to limited surveillance. Enhancing screening efforts—particularly among high-risk groups such as pregnant women—and advancing research are critical for effective hepatitis control. Improved diagnostic capabilities and targeted vaccination strategies may further reduce the associated risks, particularly in endemic regions [42] (Table 1).

### 3.2. Risk Factors of Hepatitis in Saudi Arabia

In Saudi Arabia, the transmission and prevalence of hepatitis are shaped by distinct transmission routes and associated risk factors. HAV and HEV spread mainly via the fecal–oral route, with poor sanitation, contaminated food or water, and travel to endemic areas increasing the risk. The risk factors encompass both socioeconomic determinants and behavioral influences [43].

#### 3.2.1. Socioeconomic Factors

Socioeconomic factors significantly influence hepatitis prevalence. Lower-income and rural communities experience higher rates of HAV and HEV, primarily due to inadequate access to clean water and sanitation. Alshwailem et al. reported an HAV seroprevalence of 50% among children from low-income households compared to 30% in high-income groups [44]. Similarly, HCV prevalence exhibits notable regional disparities, with higher rates in economically disadvantaged areas such as 1.5% in Jazan compared to more affluent regions like 0.5% in Riyadh [3] (Table 1).

The increased HCV prevalence among males is 0.4% vs. 0.2% in women, which may stem from a higher exposure to risk factors such as intravenous drug use, occupational hazards (e.g., healthcare work), and less frequent health-seeking behavior leading to undiagnosed chronic infections. Widowed and divorced individuals demonstrate a significantly elevated risk (adjusted odds ratios, OR: 7.8 and 2.07, respectively), potentially attributable to psychosocial stress, reduced access to healthcare, and increased engagement in high-risk behaviors following marital separation (Table 1). Additionally, the presence of comorbid conditions—such as chronic kidney disease (11.8%), diabetes (34.9%), and hypertension (35%)—further heightens vulnerability. These individuals often require frequent medical interventions (e.g., dialysis, injections) and may exhibit compromised immune function, thereby increasing their overall susceptibility [12].

HDV, a satellite virus requiring HBV coinfection, remains a critical but understudied complication in Saudi Arabia. Transmission mirrors HBV through unsafe injections, unsterilized medical equipment, or high-risk sexual contact; however, HDV’s aggressive course demands targeted screening in HBV-positive individuals, particularly migrants from endemic regions and people who inject drugs [3] (Table 1).

#### 3.2.2. Behavioral Factors

Behavioral and systemic factors such as weak healthcare infrastructure, inadequate harm reduction programs, poor surveillance, and insufficient public awareness campaigns continue to significantly influence hepatitis transmission patterns in Saudi Arabia. IVD use remains a major contributor, accounting for 30–50% of HCV cases. Although this figure is lower than in many developed countries, it likely reflects regional underreporting due to stigma and limited surveillance systems. The rising trend of opioid use across the Gulf Cooperation Council (GCC) countries, coupled with the absence of widespread harm reduction programs—such as needle exchange initiatives—further exacerbates the issue [45].

According to a study, despite strong national coverage of infant HBV vaccination, adult immunization rates remain suboptimal, with 321 out of 402 participants (79.9%) reporting having received the vaccine [46]. This falls short of the WHO’s 2030 elimination target. Gaps are especially pronounced among migrants, rural populations (e.g., Jazan and Najran), and older adults. Regional disparities are evident: for instance, Jazan reports a 40% higher rate of needle reuse than the national average, while in Makkah, approximately 8% of HCV cases are linked to unsafe practices during wet cupping (Hijama) performed during Hajj. Rural regions such as Al-Baha also show a 15–20% lower adult vaccination coverage compared to urban centers, and in Dammam, up to 30% of migrant populations remain unvaccinated due to logistical and financial barriers [47].

Comparative insights from neighboring countries reveal key opportunities for Saudi Arabia: while the UAE and Qatar match its high infant HBV vaccine coverage, they excel in migrant health screening. Kuwait reduced Hijama-related HCV cases by 60% after licensing clinics, and Iran saw a 45% HCV decline after certifying practitioners. Egypt’s mosque-based campaigns effectively countered sterilization myths. In Saudi Arabia, vaccine hesitancy persists—25% of refusals cite infertility fears, while others invoke religious beliefs like “Allah protects the pious”. Structural barriers, such as the kafala system, limit migrant healthcare access, driving 20% of Riyadh’s informal settlement HCV cases to unregulated providers. Beyond HBV/HCV, HDV affects chronic HBV patients, worsening liver outcomes but remaining underdiagnosed. Both demand targeted surveillance among high-risk groups (migrants, HBV carriers, and populations with poor sanitation) [48] (Table 1).

### 3.3. Hepatitis Treatment and Management in Saudi Arabia

#### 3.3.1. Hepatitis A

In Saudi Arabia, the MOH adheres to supportive care guidelines for HAV, as no antiviral treatment exists, recommending oral rehydration (WHO-ORS) for mild cases, IV fluids (0.9% saline with dextrose) for dehydration, 48 h liver function monitoring (ALT/AST, bilirubin) for high-risk patients, and low-fat, high-carb nutritional support to reduce liver stress [49]. Key initiatives like the Municipal Water Safety Initiative (2018–2025)—which expanded water treatment plants from 120 to 185 (2015–2021), reducing contamination-linked cases by 58% and $2.1 billion in sanitation spending (45% HAV decline), alongside the National School Vaccination Program (98% coverage, preventing 1200+ annual cases)—have been pivotal in curbing transmission [50] (Table 1).

#### 3.3.2. Hepatitis B

In Saudi Arabia, most chronic hepatitis B (CHB) cases are identified at the primary care level, though only patients with chronic active HBV are typically referred to specialists. Some healthcare centers also monitor inactive carriers [51]. Saudi Arabia’s HBV care model faces significant gaps, with only 15.7% of physicians considering primary care suitable for management. Public healthcare delivers 89% of HBV treatments (mainly nucleotide analogs), while private sector access is largely confined to major cities like Riyadh and Jeddah.

Of an estimated 260,000 chronic CHB cases, only 14% were diagnosed in the past decade, with just 14% of those (5330 out of 37,440) receiving treatment—though not all require long-term therapy. Treatment guidelines (SASLT/WHO/EASL/AASLD) recommend antivirals (tenofovir or entecavir) for HBV DNA >2000 IU/mL with elevated ALT, significant fibrosis (F2^+^), cirrhosis, or high-risk groups (e.g., immunocompromised or co-infected patients). Acute HBV typically receives supportive care, with antivirals reserved for severe cases (INR > 1.5, bilirubin > 10 mg/dL or prolonged symptoms), while chronic cases are treated proactively. Despite alignment with global standards, disparities persist, particularly in marginalized populations [52] (Table 1).

#### 3.3.3. Hepatitis C

The management of HCV in Saudi Arabia has significantly advanced with the adoption of pan-genotypic direct-acting antivirals (DAAs), which offer cure rates exceeding 95% SVR12 (sustained virologic response at 12 weeks) and are shorter, safer, and more tolerable than previous interferon-based regimens. Central to this transformation is the Saudi National Hepatitis Program (SNHP), launched in 2018, which provides free access to first-line DAAs—glecaprevir/pibrentasvir (8 weeks) and sofosbuvir/velpatasvir (12 weeks)—with ribavirin added for complex cases such as decompensated cirrhosis or HIV co-infection. Between 2018 and 2020, the SNHP screened 5 million individuals, identifying 12,000 HCV-positive cases and achieving a 92% linkage-to-care rate. By 2023, over 50,000 patients had been treated, maintaining a 95% SVR12 success rate (Table 1). This linkage rate exceeds those in many low- and middle-income countries (e.g., ~70% in India) but remains just below Egypt’s near-universal access [30].

Saudi Arabia’s elimination strategy also includes targeted micro-elimination initiatives. Innovations like point-of-care RNA testing are helping reduce diagnostic delays in rural areas, while task-shifting allows expanded treatment delivery through primary care centers. Research by institutions such as KAIMRC further supports these efforts by analyzing resistance patterns to optimize therapy [24].

#### 3.3.4. Hepatitis D

The management of HDV in Saudi Arabia remains complex and constrained by limited therapeutic options. Pegylated interferon-alpha (PEG-IFNα) has long been the standard treatment, though it offers modest efficacy, achieving a sustained virologic response in only 29% of patients, and is associated with high relapse rates approaching 50% [53]. While newer agents such as bulevirtide, an entry inhibitor with promising virologic response rates of 71–76% in clinical trials, have gained regulatory approval in regions like the EU and Russia, access to these therapies in Saudi Arabia remains limited.

The MOH guidelines recommend HDV RNA testing for HBV patients, but treatment options remain limited to PEG-IFNα. The national HBV vaccination program (since 1989) drastically reduced HBV (and thus HDV) prevalence. Strict blood donation screening and public health campaigns also contributed. Current efforts include enhanced HBV/HDV surveillance, specialized hepatology clinics, and participation in global trials for new antivirals (e.g., bulevirtide). The Saudi Liver Association promotes awareness, but access to advanced therapies remains limited compared to Western countries [54,55] (Table 1).

#### 3.3.5. Hepatitis E

For acute HEV infection, treatment remains supportive (hydration, rest, and avoidance of hepatotoxic agents), similar to HAV. However, chronic HEV, particularly in immunocompromised patients (organ transplant recipients, HIV-positive individuals, or chemotherapy patients), requires antiviral therapy. Ribavirin (600–1000 mg/day for 3 months) is the primary treatment, demonstrating a 78–83% SVR (sustained virologic response) in European studies, though with a risk of hemolytic anemia (18%) requiring dose adjustments. Pegylated interferon (PEG-IFN) is an alternative but carries significant side effects, limiting its use [56] (Table 1).

Saudi Arabia’s HEV control efforts include water sanitation, enhanced surveillance in high-risk groups (migrants, transplant recipients, and chemotherapy patients), and ribavirin availability in tertiary hospitals, alongside European collaborations for treatment protocols. Challenges remain, such as underdiagnosis due to limited PCR testing, no national HEV registry, and a lack of targeted screening in transplant centers. Future priorities include establishing a registry, routine screening in high-risk settings, and exploring new antivirals beyond ribavirin [40].

#### 3.3.6. Government and Non-Governmental Organizations (NGOs) Initiatives

Between 2018 and 2023, the SNHP screened approximately 20% of the population, significantly contributing to a reduction in HCV prevalence—from 0.4% among adults to 0.1% in children. The program implemented extensive screening campaigns across public spaces such as malls, universities, and mosques. Complementing these efforts, the Saudi Centers for Disease Control and Prevention (CDC) launched the “Know Hepatitis” data-tracking initiative, while King Faisal Specialist Hospital advanced elimination strategies in dialysis units, including large-scale public screening, data-driven tracking systems, targeted high-risk group interventions (e.g., dialysis patients), and high-efficacy antiviral treatments (95%+ cure rates), aligning with WHO 2030 elimination goals [57].

Saudi Arabia’s integrated and large-scale approach has achieved HCV cure rates exceeding 95%, while HBV prevalence has declined to 1.3%, positioning the country on track to meet the World Health Organization’s target of HCV elimination by 2030. While SNHP and other initiatives have focused heavily on HCV and HBV, there are no major nationwide programs targeting other hepatitis types (such as HAV, HDV, or HEV). This is likely due to their lower prevalence in Saudi Arabia or less severe long-term health impacts compared to HCV and HBV, which are prioritized for elimination under WHO goals. However, challenges remain in ensuring equitable access to hepatitis care. Treatment availability in rural regions remains 35% lower than in urban centers, and migrant populations experience a 30% lower treatment uptake, often due to structural and logistical barriers [58].

### 3.4. Successful Policies and Interventions to Prevent and Control Hepatitis in Saudi Arabia

#### 3.4.1. Vaccination Programs

Since 1990, Saudi Arabia has implemented a universal HBV vaccination program under its Expanded Program on Immunization (EPI), incorporating the HBV vaccine into the national immunization schedule for infants. The program was officially approved in 1989 and fully launched the following year. Vaccination coverage for HAV in the region is 97.2%, according to MoH (2023) [9]. A key component involves administering the first HBV vaccine dose within 24 h of birth, a strategy proven to significantly reduce mother-to-child transmission (MTCT). Without appropriate prophylaxis, infants born to mothers who are positive for both HBsAg and hepatitis B e-antigen (HBeAg) face a 70–90% risk of perinatal HBV transmission [59].

Al-Faleh’s study further supports these findings, reporting a 77% overall seroconversion rate in 4087 children aged up to 12 years. Children vaccinated at birth showed a slightly higher seroconversion rate (77%) compared to those vaccinated later at school entry (71%). Long-term immunity was also documented: eight years after completing the three-dose regimen, 65% of children retained anti-HB titers above 10 IU/L, while 28% maintained titers above 100 IU/L [36]. Saudi Arabia’s HBV vaccination program has achieved a 98% newborn coverage and a 90% decline in childhood HBV incidence since 1989. Moreover, a 2023 study reported that the universal mass vaccination program in the region reduced the incidence rate of HAV. School-based campaigns (95% coverage) and healthcare worker mandates (97% coverage) also reduced occupational transmission by 85% (Table 1). By 2015, these efforts drove national HBsAg prevalence below 1%, demonstrating the success of targeted strategies [60].

On the other hand, the HAV vaccination program was introduced into Saudi Arabia’s National Immunization Program in 2008, targeting children to reduce the incidence of the disease. The program has been successful, contributing to a significant decline in hepatitis A cases across the country. According to health reports, vaccination coverage has reached over 90% in some regions, leading to a drastic reduction in outbreaks and related hospitalizations. The success of the program is attributed to high public awareness, efficient vaccine distribution, and integration with routine childhood immunizations [61] (Table 1).

#### 3.4.2. Screening and Early Diagnosis Programs

In addition to widespread vaccination, Saudi Arabia has prioritized national screening and early detection initiatives to identify and treat HBV and HCV infections at an early stage. A key milestone was the 2014 nationwide prenatal screening program, which ensured that 99% of pregnant women were tested for HBV, facilitating timely antiviral therapy and significantly reducing the risk of mother-to-child transmission. Premarital screening efforts have also contributed to early detection, with studies reporting hepatitis B surface antigen (HBsAg) positivity rates ranging between 1.3% and 1.7% (Table 1). However, these figures may underestimate the national burden due to a sampling bias toward younger, healthier individuals.

Earlier regional studies, conducted before the implementation of universal blood screening and the full rollout of vaccination programs, revealed much higher HBsAg prevalence rates, ranging from 4.1% to 17.3% across various provinces. The effectiveness of preventive strategies is further highlighted by longitudinal data: two studies of couples attending fertility clinics a decade apart reported a significant decline in HBV prevalence, from 4.7% during 2002–2005 to 1.7% in 2023. This marked reduction underscores the success of Saudi Arabia’s screening and prevention policies [62].

#### 3.4.3. Knowledge and Awareness Gaps

Public awareness has been a key component of Saudi Arabia’s hepatitis control strategy. A 2017 nationwide study reported that 91% of participants were generally aware of hepatitis; however, only 54% demonstrated a good understanding of HBV, and just 64.5% correctly identified it as a viral infection. Regional studies further highlight gaps in public knowledge [63]. For instance, in Taif, 80.4% of respondents had heard of HBV, yet in-depth understanding remained limited. Multivariable analysis from this study identified higher education, employment in the medical sector, and a monthly income exceeding SAR 5000 as significant predictors of HBV knowledge [64]. Knowledge of HBV transmission routes (e.g., sexual contact and mother-to-child) is frequently low, and understanding of the HBV vaccination schedule is incomplete, leading to suboptimal immunization coverage [65,66,67]. Concerning HCV, many incorrectly believe HCV can be transmitted through casual contact (e.g., kissing or sharing utensils) or that an HCV vaccine exists [66,68,69].

High-risk groups, such as people who inject drugs (PWID), often lack detailed knowledge about HCV transmission and the availability of curative direct-acting antivirals (DAAs), compounded by structural barriers like stigma and limited healthcare access [65,70,71]. Indigenous, incarcerated, and migrant populations have understudied awareness levels despite high exposure risks, with few targeted interventions available [69,72,73]. Pregnant women and healthcare providers in endemic regions sometimes have gaps in knowledge regarding HBV mother-to-child transmission prevention, including the timing of neonatal vaccination and immunoglobulin administration [74].

Physicians and other healthcare workers exhibit varying levels of knowledge with persistent gaps. Some providers mistakenly believe an HCV vaccine exists or underestimate non-parenteral transmission routes (e.g., sexual or tattooing for HCV) [69,74]. Screening practices are inconsistent, often due to unfamiliarity with guidelines or systemic barriers [74,75]. Key knowledge deficits exist in HBV management, including newborn immunization protocols and HBV reactivation risks during immunosuppressive therapy [75,76]. Paramedical and support staff often have lower awareness and vaccination rates than physicians, limiting their role in patient education [77].

These findings point to disparities in awareness based on socioeconomic and educational background. Notably, 19.1% of participants had only secondary education, suggesting the need for simplified educational resources that use accessible language and visual aids. Public health campaigns could also target self-employed individuals—who constituted 35.3% of the surveyed population—through outreach in community centers and workplaces. Moreover, providing free or subsidized vaccinations for lower-income groups earning between SAR 0 and 10,000 could help mitigate financial barriers to prevention. By leveraging diverse communication platforms, promoting community engagement, and designing culturally tailored health education tools, Saudi public health authorities can significantly improve the public understanding of HBV (Table 1). Such efforts, particularly in high-density urban areas like Makkah, are essential for enhancing vaccine uptake, reducing transmission, and improving long-term health outcomes [78].

### 3.5. Challenges and Barriers

#### 3.5.1. Social Stigma in Patients

Stigma remains a major barrier to HBV diagnosis and treatment in Saudi Arabia, driven by the fear of infection, shame, social rejection, and economic consequences like job insecurity. A national survey found that 62% of patients delayed testing due to fear of discrimination, while 45% concealed their status even from family. Gender disparities worsen the issue, with men 3.2 times more likely to seek treatment than women (*p* < 0.01), and 58% of female patients reporting discouragement by physicians due to pregnancy concerns. Stigmatized patients show 40% lower treatment adherence and are 2.5 times more likely to progress to cirrhosis (OR = 2.48; 95% CI: 1.67–3.69). Additionally, 34% of patients believed HBV was divine punishment, delaying care. Combating stigma requires public education, healthcare provider training, and community engagement to improve outcomes [65].

#### 3.5.2. Economic Impact of Antiviral Therapy for HBV

In Saudi Arabia, entecavir (ETV)—the most commonly prescribed nucleos(t)ide analog (NUC) in neighboring countries like the UAE (73% of prescriptions due to its safety and low resistance)—is provided free to citizens through government-funded healthcare, ensuring equitable HBV treatment access. However, this imposes a significant economic burden, with the 2022 healthcare expenditure reaching SAR 147 billion (USD 39 billion), over 15% of total government spending, driven partly by lifelong antiviral therapies like ETV and tenofovir alafenamide (TAF). To address this, Vision 2030 reforms aim to enhance efficiency by streamlining protocols, prioritizing cost-effective regimens, and promoting private sector engagement, alongside early detection, vaccination, and prevention programs to sustain high-quality HBV care while ensuring long-term healthcare system sustainability [79].

### 3.6. Future Directions to Control and Prevent Hepatitis in Saudi Arabia

#### 3.6.1. Enhancing Viral Hepatitis Prevention and Control Strategies

Prevention strategies should focus on expanding vaccination programs, particularly ensuring universal HBV vaccination coverage, including catch-up campaigns for high-risk groups such as healthcare workers, migrants, and prisoners. Additionally, introducing routine HCV screening for pregnant women and high-risk populations could significantly reduce transmission rates. Public awareness campaigns are also crucial to reduce stigma and encourage testing, with hepatitis education integrated into school health programs.

Blood and injection safety must be reinforced through the strict enforcement of screening protocols and sterile medical practices. On the treatment front, scaling up access to DAAs for HCV and antiviral therapy for HBV is essential. Establishing specialized hepatitis treatment centers in rural areas would improve accessibility, particularly for underserved populations. Strengthening surveillance systems through real-time electronic reporting and regular seroprevalence studies will help monitor progress and identify emerging trends [80].

#### 3.6.2. Future Research and International Collaboration Priorities

Future research should focus on three key areas. First, epidemiological studies are needed to better assess HBV and HCV prevalence in high-risk groups (e.g., intravenous drug users, expatriate workers) and to evaluate the role of migration in transmission. Second, treatment optimization research should investigate HBV functional cure strategies, HCV reinfection rates post-treatment, and the cost-effectiveness of decentralized testing models. Third, innovation in prevention and therapy remains essential, including the development of an HCV vaccine and improved HBV immunotherapies [44].

In addition, HDV and HEV are critically understudied in Saudi Arabia. HDV affects an estimated 7.7% of chronic HBV patients and accelerates disease progression, yet surveillance and treatment remain limited. Similarly, HEV continues to pose outbreak risks, particularly during mass gatherings such as Hajj, but comprehensive national data and screening protocols are lacking. Expanding epidemiological studies and integrated surveillance for HDV and HEV should be prioritized to close these gaps [70]. In addition, there is a clear need for comparative analyses with neighboring countries, particularly to evaluate differences in morbidity and vaccination coverage. Within Saudi Arabia, monitoring the prevalence of viral hepatitis across different population groups and genders over time is also essential to identify disparities, guide tailored interventions, and assess progress toward elimination goals [73].

Furthermore, international collaboration is pivotal for hepatitis elimination. Joint research and data sharing with organizations such as the WHO, the Global Hepatitis Network, and GCC partners can support harmonized regional strategies. Capacity-building initiatives, including professional exchanges with leading treatment centers in the U.S., EU, and Egypt, can strengthen local expertise. Policy alignment with WHO guidelines will help ensure Saudi Arabia stays on track with elimination targets [81].

Evidence supports these efforts: countries with robust international collaboration achieved a 50% faster reduction in HCV incidence, while the Saudi Ministry of Health’s partnerships with Egypt boosted local HCV testing rates by 25% in 2022 [71].

## 4. Conclusions

This review highlights that despite notable progress in vaccination and treatment programs, viral hepatitis continues to pose a significant public health challenge in Saudi Arabia. While immunization and antiviral therapies have reduced prevalence, persistent issues such as diagnostic delays, healthcare disparities, and social stigma hinder elimination efforts. The study acknowledges limitations, including reliance on facility-based data and regional reporting inconsistencies, which emphasize the need for stronger surveillance systems. Moving forward, achieving WHO 2030 elimination targets will require a sustained commitment to evidence-based policies, health system strengthening, and innovative strategies. Future priorities should focus on targeted screening, optimized care delivery models, and enhanced global collaboration, underscoring the necessity of a comprehensive, multisectoral approach for sustainable hepatitis control in the Kingdom.

## Figures and Tables

**Table 1:** Epidemiology, risk factors, treatment, and prevention strategies for hepatitis viruses.

Virus	Transmission	Regional Trends	Risk Factors	High-Risk Groups	Treatment	Prevention	References
HAV	Fecal–oral	Intermediate endemicity; higher in less developed regions	Contaminated water/food, poor sanitation, and travel	Children, adolescents, and travelers	Supportive care: oral/IV rehydration, liver function monitoring	HAV vaccine; school-based campaigns	[1,2]
HBV	Blood-borne, vertical, and sexual	Declining nationally; regional hotspots (Qunfudah, Jeddah)	Blood transfusions, unsafe injections, and vertical transmission	Neonates of HBV-positive mothers, healthcare workers, and migrants	Antivirals: tenofovir, entecavir; monitoring inactive carriers	Universal infant vaccination; prenatal and premarital screening	[3,4,5,6,7,9,10]
HCV	Blood-borne, IVDU, and unsafe procedures	Regional variation; Jeddah highest burden	Unsafe injections, IVDU, and hemodialysis	Adults ≥ 45, males, PWID, and migrants	DAAs: Glecaprevir/Pibrentasvir, Sofosbuvir/Velpatasvir; >95% SVR	No vaccine; national screening and micro-elimination programs	[1,8,11]
HDV	Requires HBV co-infection	Limited data; underdiagnosed	HBV co-infection, blood/body fluids, and sexual contact	Chronic HBV carriers, migrants from endemic areas	PEG-IFNα (29% SVR), Bulevirtide (71–76% efficacy)	Indirect via HBV vaccination; HDV testing for HBV patients	[3,7]
HEV	Fecal–oral	Outbreaks during mass gatherings; fluctuating prevalence	Contaminated water, poor sanitation, and mass gatherings	Pregnant women, immunocompromised	Supportive care; ribavirin for chronic cases (78–83% SVR)	No widespread vaccine; hygiene, water sanitation	[2]

## Data Availability

No new data were generated or analyzed in this study. This literature review is based on previously published articles and publicly available sources. All referenced studies are cited appropriately in the references section.

## Abbreviations

The following abbreviations are used in this manuscript:

SNHP	Saudi National Hepatitis Program
HAV	Hepatitis A virus
HBV	Hepatitis B virus
HDV	Hepatitis D virus
HEV	Hepatitis E virus
HCC	Hepatocellular carcinoma
IDU	Intravenous drug use
RCTs	Randomized controlled trials
WHO	World Health Organization
UAE	United Arab Emirates
HBsAg	Hepatitis B surface antigen
GCC	Gulf Cooperation Council
CHB	Chronic hepatitis B
DAAs	Direct-acting antivirals
PEG-IFNα	Pegylated interferon-alpha
PEG-IFN	Pegylated interferon
NGO	Non-Governmental Organizations
CDC	Centers for Disease Control and Prevention
HBeAg	Hepatitis B e-antigen
PWID	People who inject drugs
TAF	Tenofovir alafenamide
